# Out-of-hospital cardiac arrest in Bahrain: National retrospective cohort study

**DOI:** 10.1016/j.resplu.2024.100778

**Published:** 2024-09-14

**Authors:** Feras Husain Abuzeyad, Yasser Chomayil, Moonis Farooq, Hamid Zafar, Ghada Al Qassim, Emad Minwer Saad Albashtawi, Leena Alqasem, Naser Mohammed Ali Mansoor, Danya Adel AlAseeri, Ahmed Zuhair Salman, Muhammad Murad Ashraf, Maryam Ahmed Shams, Faisal Sami Alserdieh, Mustafa Ali AlShaaban, Abdulla Fuad Mubarak

**Affiliations:** a Department of Emergency Medicine , Farwaniya Hospital, Farwaniya, Kuwait; b Department of Emergency Medicine, King Hamad University Hospital, Building 2345, Road 2835, Block 228, P. O. Box 24343, Busaiteen, Bahrain; c Department of Emergency Medicine, Queen Elizabeth Hospital, London, United Kingdom; d Pediatric Emergency , Military Hospital-Royal Medical Services, Bahrain Defence Force, Riffa, Bahrain; e National Ambulance Centre, Ministry of Interior, Bahrain; f Authority for Medical Responsibility, Kuwait; g Department of Emergency Medicine, Salmaniya Medical Complex, P.O. Box 12, Manama, Bahrain; h Department of Emergency Medicine, Military Hospital-Royal Medical Services, Bahrain Defence Force, Riffa, Bahrain; i Royal College of Surgeons in Ireland – Bahrain, Building No. 2441, Road 2835, Busaiteen 228, Bahrain

**Keywords:** Out of hospital cardiac arrest, Emergency medical services, Cardiopulmonary resuscitation, Bystander CPR, OHCA registry

## Abstract

**Aim:**

There is limited research on Out-of-hospital cardiac arrest (OHCA) in the Gulf Cooperation Council (GCC) and especially in Bahrain. This is the first study to describe the incidence, characteristics, and outcomes of OHCA in Bahrain.

**Methods:**

This was a retrospective national observational study on OHCA patients in Bahrain using the Utstein framework for resuscitation. Data was collected between 1st July 2022 to 30th June 2023 from the electronic medical records of the only three governmental hospitals emergency departments (EDs) and National Ambulance (NA).

**Results:**

The annual incidence of OHCA attended by (Emergency Medical Services) EMS was nearly 21 per 100,000 population. The majority were males (n = 228, 68.8 %) with median age of 65 years (IQR=49–78). Most OHCA cases were witnessed (n = 265, 81 %), with (n = 247, 76 %) happened at home/residence. Rates for bystander CPR was low (n = 122, 36.8 %) and bystander automated external defibrillator (AED) was not performed in any of the cases. The OHCA cases transported by the NA was (n = 314, 94.8 %), with median response time of 9 min (IQR=7–12). However, only (n = 20, 6.0 %) were witnessed by EMS, and (n = 7, 2.1 %) received EMS defibrillation for shockable rhythms. First monitored rhythms included shockable rhythm in (n = 28, 8.5 %) versus non-shockable rhythm in (n = 303, 91.5 %). In the EDs, return of spontaneous circulation was achieved in (n = 60, 18.1 %) cases. But survival rate to hospital discharge at 30-day was (n = 4, 1.2 %) and survival rate to hospital discharge with good neurological outcomes was (n = 0, 0 %). Conclusion: In Bahrain the estimated annual incidence of OHCA is 21 individuals per 100,000 population, with a very low survival rate. Solutions should focus on community-level CPR and AED training, evaluating OHCA care provided by EMS, and establishing OHCA registry.

## Background/introduction

Worldwide, out-of-hospital cardiac arrest (OHCA) is a key healthcare problem, with a huge financial burden on the healthcare system. For example, in the United States (US), OHCA increased the total annual economic productivity loss to $11.3B and the lifetime economic productivity loss to $150.2B in 2018.^1^ The average international incidence of OHCA is 55 per 100,000 person-years.^2^ OHCA is an emergency condition that is time-dependent and the survival rate decreases with every-minute delay in cardiopulmonary resuscitation (CPR),^3^ and the outcomes are affected by multiple variables including the demographics, etiology of arrest, comorbidities, initial arrest rhythm, arrest location, bystander CPR, and EMS interventions.^4^ OHCA survival will depend on the American Heart Association (AHA) chain of survival which consists of six links: early recognition and activation of EMS, early CPR, rapid defibrillation, and advanced life support by EMS, post-cardiac arrest care, and recovery therapy.^5^ These concepts are drawn around the Utstein Survival Formula which link (1) medical science with (2) educational efficiency and (3) local implementation programs to improve the OHCA survival by improving one or more of the related domains.^6^ The formation of OHCA registries was based on the incorporation of the Utstein-templet variables which allows patients data to be collected, analysed, and studied, for measuring the incidence, variables, outcomes, quality control, and benchmarking different regions. The number of OHCA registries is increasing and spreading on a national, sub-national, and regional levels worldwide with very valuable benefits.^7^

Unfortunately, Bahrain does not have a national registry for OHCA patients, with no data on its incidence, characteristics, or outcomes. A scoping review on OHCA in the Gulf Cooperation Council (GCC) countries had shown that it is under researched with no literature identified for Bahrain, and there is a great necessity to establish dedicated organisations to develop and coordinate policies to address OHCA, and this include public education, bystander response, and a registry system.^8^ To our knowledge, this is the first national study to investigate OHCA in Bahrain using Utstein framework.

## Method

### Design and setting

This is the first national multicentre retrospective observational study of all patients with OHCA who presented to the governmental hospitals emergency departments (EDs) between 1st July 2022 to 30th June 2023 in the Kingdom of Bahrain.

Data was collected on OHCA patients using constructed data collection form based on the latest templates of the Utstein resuscitation guidelines.^9^ Data was obtained from the patient electronic medical records and the CPR forms in the three major governmental EDs serving the country. In addition, for OHCA patients resuscitated and transported by the National Ambulance (NA), the pre-hospital care data were retrieved from the patient care reports.

### EMS system in Bahrain

The Kingdom of Bahrain has a land area of 786.5 Km^2^ and it is divided into four governances: The Capital, Muharraq, Northern, and Southern which are inhabited with a population of 1,504,365 making it one of the highest populated countries with a population density of 1912.73 people/Km^2^.^10^

In the Kingdom, the emergency medical services (EMS) are provided by the NA which is a governmental directorate under the ministry of interior that provides a unified and central ambulance services.^11^ The EMS system follows the Anglo-American model and is based on single-tier system run by paramedics and ambulance nurses providing both basic life support (BLS) and advanced cardiac life support (ACLS) at the scene as needed.^12^ To ensure rapid and fast response the NA receives emergency phone calls through “999” which are filtered and evaluated using the priority dispatch system (PDS) which then dispatches an ambulance from one of the 13 dispatch stations that cover the whole country.^11^ There are only three governmental hospitals in the kingdom including Salmaniya Medical Complex (SMC), Bahrain Defence Force (BDF) Hospital, and King Hamad University Hospital (KHUH) and all receive patients through the NA to be managed in their EDs.^13^ The NA adopted standardized dispatch process for assistance through the PDS protocols from “ProQA”. The PDS software protocols allow for caller interview, codes determination assignment, and delivery of post-dispatch and pre-arrival directions. NA prehospital providers retain valid courses in BLS, ACLS, paediatric advanced life support (PALS), and pre-hospital trauma life support (PHTLS) and are licensed by the National Health Regulatory Authority (NHRA) as paramedics or ambulance nurses and they operate under written clinical protocols and online medical directions.^11^

In the field, the NA prehospital care providers are not allowed to withhold CPR or terminate resuscitation for OHCA patients unless there are clear signs of death. For this reason, NA created specific clinical policy for its staff to assess the patients in cardiac arrest and decide whether CPR is to be started or withheld. The decision procedure is based on respiratory, cardiac, and neurological assessment criteria. If the NA team started the CPR at the scene, it will be continued till arrival to the closest hospital and the case will be handed over to the ED medical team. In some Asian countries, the field termination of resuscitation is prohibited and it is mandatory to resuscitate and transport all OHCA patients to the hospitals.^14^, ^15^, ^16^

### Participants (inclusion/exclusion criteria)

The study population included only adults with age ≥ 18 years with presumed medical OHCA. OHCA is described as the cessation of the cardiac mechanical activity which is associated with lack of signs of circulation that occurs outside the hospital premises.^9^

Patients with non-cardiac etiology of arrest such as trauma, suicide, drug overdose, asphyxia, drowning, electrocution were excluded. Also, we excluded cases which were < 18 years, pronounced dead upon arrival at the scene, or their data were incomplete.

### Outcomes

The study examined several outcomes as per (table-3). However, the primary outcome measured was 30-day survival to hospital discharge and good neurological outcome on hospital discharge. Good neurological outcome was defined as cerebral performance category (CPC) scale category 1 or 2.^17^

### Ethical approval

The four institutions received the study approval; SMC from the the Research Committee for Government Hospitals (serial no: 57030623), BDF Hospital from the Research & Research Ethics Committee (reference number: BDF/R&REC/ 2023–668), KHUH from the institutional review board (reference number: 23–610), and the NA administration (letter number: RAA/2/43/23/411).

### Statistical analysis

Descriptive statistics was used to compute the frequency and percentages for the categorical data. Mean + SD and median was computed for continuous variables. All analysis was performed using SPSS v 26.0 (IBM, New York, USA).

## Results

A total of 376 EMS-treated OHCA cases were reported during the study period of which 23 (14 males and 9 females) were paediatric cases and hence were excluded from the study. Also, we excluded eight trauma cases, four cases of drug overdose, three cases of drowning, and seven cases with more than 50 % incomplete information. Hence 331 patients were included in the study. The incidence of those attended by EMS was about 21 individuals per 100,000 population. The mean age of 331 OHCA patients included in the study was 63.02 years (SD 18.51), median age of 65 (49–78). Higher incidence was noted among males (228, 68.8 %) as compared to females (103, 31.1 %). The majority of the OHCA cases were reported by bystander (265, 80.6 %), 46 (13.8 %) were unwitnessed and 20 (6.0 %) were witnessed by EMS. About 74.6 % of the OHCA cases reported home/residence as location of arrest followed by 13.8 % reported from public places, 4.5 % from industrial/workplace and 3.0 % from assisted living/ nursing home. In less than half cases (122), bystanders attempted compressions (36.8 %) and in 12 cases (3.6 %) bystanders attempted both compression and ventilation. AED was used by EMS in only 17 (5.1 %) cases, 7(2.1 %) with shock delivered and 10 (3.0 %) without shock delivered. Majority of the cases (271, 81.8 %) reported to have 'asystole' as first monitored rhythm. Hypertension and diabetes mellitus were the major comorbidity reported among 53.4 % and 50.1 % cases respectively. Other comorbidities reported were dyslipidemia (31.1 %), IHD (26.2 %) and CKD (10.8 %). (Table 1).

**Table 1:** Characteristics of out-of-hospital cardiac arrest patients (N=331).

**Age**	
Mean ± SD	63.02 ± 18.51 (18- 98)
Median	65 (49-78)

**Gender, n (%)**	
Male	228 (68.8%)
Female	103 (31.1%)

**Witnessed arrest, n (%)**	
Bystander witnessed	265 (80.6%)
EMS witnessed	20 (6.0%)
Unwitnessed	46 (13.8%)

**Location of arrest, n (%)**	
Home/residence	247 (74.6%)
Public place	46 (13.8%)
Industrial/workspace	15 (4.5%)
Assisted living/nursing home	10 (3.0%)
Other	11 (3.3%)
Not recorded	2 (0.6%)

**Bystander response, n (%)**	
Bystander CPR: compression only	122 (36.8%)
Bystander CPR: compression and ventilations	12 (3.6%)
no bystander CPR	197 (59.5%)

**AED use by bystander/ paramedics, n (%)**	
AED used, shock delivered (by EMS)	7 (2.1%)
AED used, no shock delivered (by EMS)	10 (3.0%)
AED not used (by EMS)	314 (94.8%)
AED used (by bystander)	0 (0%)

**First monitored rhythm, n (%)**	
VF	27 (8.15%)
pulseless VT	1 (0.3%)
PEA	32 (9.66%)
asystole	271 (81.87%)

**Independent living, n (%)**	
Yes	122 (36.8%)
No	207 (62.5%)
unknown/not recorded	2 (0.6%)

**Past Medical History, n (%)**	
Diabetes Mellitus	166 (50.1%)
Hypertension	177 (53.4%)
Dyslipidemia	103 (31.1%)
Smoking	24 (7.2%)
IHD	87 (26.2%)
Malignancy	12 (3.6%)
CKD	37 (10.8%)
Connective tissue disorder	3 (0.9%)
Obesity	22 (6.6%)
VAD	0 (0.0%)
Cardioverter-defibrillator	1 (0.3%)
Presence of STEMI post ROSC	5 (1.5%)

Three hundred and fourteen patients (94.8 %) were brought in by the EMS and 17 (5.13 %) were brought in by private transport. The NA had a median response time of 9 min (IQR=7–12) and the mean ± SD was 10.00 ± 4.78 min. The defibrillation time was recorded only in 28 patients (8.4 %) with a median time for the recorded cases of 20 min (IQR=13–30) and the mean ± SD was 22.47 ± 12.02 min. (Table 2).

**Table 2:** EMS involvement phase.

**Mode of arrival, n (%)**	
EMS	314 (94.8%)
Private	17 (5.13%)

**Response time**	
Recorded, n (%)	314 (94.8%)
Not applicable (NA), n (%)	17 (5.13%)
Median time for recorded cases (minutes) Median (IQR)	9 (7– 12)
Mean±SD (minutes)	10.00 ± 4.78

**Defibrillation time**	
Recorded, n (%)	28 (8.4%)
Not applicable (NA), n (%)	303 (91.8%)
Median time for recorded cases (minutes)	20 (13-30)
Mean±SD (minutes)	22.47 ± 12.02

**Drugs given, n (%)**	
Adrenaline	330 (99.7%)
Amiadarone	26 (7.8%)

Sixty patients (18.1 %) achieved ROSC in the EDs and they survived to hospital admission. Only 4 patients (1.2 %) survived to hospital discharge at 30-day and none had good neurological outcome on hospital discharge. (Table 3).

**Table 3:** Outcome of out-of-hospital cardiac arrest patients (N=60).

Outcome	N (%)
Any ROSC	60 (18.1 %)
ROSC before arrival to ED (prehospital)	0 (0 %)
ROSC in ED	60 (18.1 %)
Survival to hospital admission	60 (18.1 %)
ED transfers to other hospital after ROSC	0 (0 %)
30-day survival or survival to hospital discharge	4 (1.2 %)
Good neurological outcome on hospital discharge	0 (0 %)

## Discussion

This is the first national study in Bahrain to investigate OHCA using Utstein framework. The Utstein style had been commonly used as it enable researchers to collect a wide range of data on OHCA patients and it facilitates the comparison between different systems which use it.^18^ The study showed an approximate annual incidence of EMS attended OHCA of about 21 per 100,000 population. In GCC countries, Saudi Arabia reported an incidence of 23 per 100,000 population,^19^ while Qatar had an incidence of 23.5 per 100,000 population.^20^

The International Liaison Committee on Resuscitation (ILCOR), in its first report had an annual incidence of EMS treated OHCA ranged between 30.0 and 97.1 per 100,000 population.^21^

The mean age of OHCA patients was 63.02 years (SD 18.51) with a median age range between 49 to 78 years, and 68.6 % were males. About 81 % were bystander witnessed arrests, and only 6 % were EMS witnessed. The location of the arrest showed about 75 % occurred at home/residence, 14 % occurred at public places, and 5 % at work place. However, only 36.8 % and 3.6 % received compression-only and compression-ventilations by bystander respectively, with bystander automated external defibrillator (AED) use of 0 %. The ILCOR reported a rate of 37–69.8 % for bystander witnessed OHCA, with 51.6 % to 85.3 % happened at home.^21^ In the Pan Asian Resuscitation Outcomes Study (PAROS), the rates of bystander CPR ranged between 10.5 – 40.9 % with < 1 % received bystander defibrillation.^22^ While in Europe, the bystander CPR and bystander AED use were ranging from 13 % to 83 % and 3.8 % to 59 % respectively.^23^

The OHCA cases transported by the NA counted for about 95 %, with median response time of 9 min (IQR=7–12), which is very close to the Qatari study with median response time of 8.72 min (IQR=6.8–11.8),^20^ but is much lower when compared to Saudi Arabia with a reported median response time of 13 min (IQR=9–18).^19^. In Asia, Taiwan and Malaysia had the shortest and longest response time with a median response time of 5.2 min (IQR=4.1–7.0) and 17.4 min (IQR=12.0–24.2) respectively.^22^ The regional registry Saving Hearts in Arizona Registry & Education (SHARE) in US reported the shortest response time of 5 min (IQR=4–7).^21^

Many EMS agencies use the operational defibrillation response interval (≤8 min for at least 90 % of cardiac arrest cases) as the optimal time for scene defibrillation with no well-supported evidence, and we should differentiate it from the clinical defibrillation response interval which reflects the period from the victim’s collapse till first shock is delivered.^24^ To improve survival, EMS agencies should consider using an operational defibrillation response interval of 5–6 min 90 % of the time as a target.^24^

In the study, the first monitored rhythms were non-shockable rhythm in about 91 %, and shockable rhythm in nearly 8 % with only about 2 % who received EMS defibrillation in the field with a median time of 20 min (IQR=13–30). The study recorded only the operational defibrillation time with no data on the clinical defibrillation time. A retrospective study from the Korean registry of OHCA patients, looked at the clinical defibrillation time by EMS and good neurological outcomes in witnessed OHCA patients. They divided the times from collapse to first defibrillation into four groups: 1 (0–5 min), 2 (6–10 min), 3 (11–15 min), and 4 (16–60 min), and short time was associated with better rates of survival to discharge and ROSC, and improved neurological outcomes.^25^

Non-shockable rhythm is the presenting rhythm in 75–72 % of the cardiac arrest population,^3^ and several studies demonstrated temporal trend with an increase in the incidence of non-shockable rhythm presenting as the initial rhythm in OHCA adult patients particularly in residential location.^26^, ^27^, ^28^ Non-shockable rhythms can be late presentation of delayed or untreated shockable rhythms (ventricular tachycardia and fibrillation). In Japan, a shorter response from first collapse-to-CPR in witnessed OHCA was associated with a better outcome, and the proportion of asystole rhythm increased as the response time from collapse-to-CPR prolonged.^29^ The high proportion of first monitored non-shockable rhythm (91 %) could be explained by the low bystander CPR, absent bystander AED, and the long operational defibrillation time by EMS. However, this need to be investigated in another study.

The study found the three commonest prior comorbidities; hypertension, diabetes, and dyslipidaemia with 53.4 %, 50.1 %, and 31.1 % respectively. In general, pre-arrest comorbidity is associated with reduced survival and poorer neurological outcomes following OHCA, especially with diabetes as a prior comorbidity.^30^

In the study, the survival rates to hospital admission and discharge at 30-day were 18.1 % and 1.2 %. Unfortunately, none had good neurological outcome on hospital discharge. Studies from Saudi Arabia and Qatar calculated a survival rates to hospital discharge of 2.9 % and 8.1 % respectively and only < 0.5 % and 5.3 % respectively had good neurological outcomes at discharge.^19^, ^20^ In PAROS, the reported rates of survival to discharge and the survival with good neurological outcome were 0.5–––8.5 % and 1.6 – 3 % respectively.^22^ Globally, the survival rate ranged between 3.1 % to 20.4 % and the survival rate with good neurological outcome ranged between 2.8 % to 18.2 % at 30 days of hospital discharge.^21^

The low survival rate in the study could be attributed to several factors but the most vital one at the community level is the low rate of bystander CPR and the lack of public access to AEDs. The AHA has emphasised in the chain of survival the importance of; fast recognition of cardiac arrest with early EMS notification, bystander provision of CPR and rapid defibrillation till EMS arrival.^5^ Over the past 40 years, the global survival rate for adult OHCA patients increased in patients who received bystander CPR and who were living in North America and Europe.^31^

So, to enhance OHCA survival, it is crucial to invest in national educational and training programs at multiple levels for cardiac arrest recognition, early and effective bystander CPR, and public-AED usage.^32^ Also, it is essential to invest in dispatcher-assisted CPR system as it has been proven to have a valuable effect on patient outcomes following cardiac arrest by increasing the rate of bystander CPR.^33^ The following three public-based interventions: bystander CPR training, public-AED training, and dispatcher-assisted CPR had a synergistic effect, and were associated with significant increase in bystander CPR rate, bystander AED rate, OHCA survival, and OHCA survival with good neurological outcome.^34^

Another factor for the low OHCA survival could be related to the NA operating system, as the majority of OHCA cases are managed in the field with single-tier BLS/ALS unit comprising of one paramedic and one paramedic assistant conducting the entire resuscitation with ‘scoop and run’ transport concept. Field resuscitation is an option, especially with increased evidence to support on-scene resuscitation as intra-arrest transport disturbs and reduces CPR quality severely.^35^ However, if this is considered, NA might need to deploy more EMS personnel to the scene for cardiac arrest patients. In the EMS literature there are no guidelines for the optimal number of EMS personnel required to respond for OHCA patients, and the number defers from region to region as it is related to the EMS organizational structure. Few studies suggested an EMS crew of at least 6 personnel at the scene were associated with better outcomes for OHCA.^36^, ^37^, ^38^ Increasing the dispatched EMS personnel on-scene could be a changeable factor for improving the outcomes of OHCA, as more providers can achieve high quality CPR, rapid defibrillation, and ALS interventions as needed. A simulation study examined the optimal EMS team size for OHCA resuscitation, and found the 5-personnel team had the best resuscitation performance and team dynamics, while the 2-personnel EMS team had the longest period of resuscitation interruption and the lowest chest compression fraction in manual CPR.^39^

Finally, the policymakers in Bahrain should consider establishing national OHCA registry, and look at the various potential barriers to improve OHCA resuscitation care system and outcomes.^40^

### Limitations

The study had few limitations. The first is related to the study design, the retrospective data collection suffered from some incomplete and missing information. The second limitation is that the private hospitals were not represented in this study and OHCA patients presented to these institutions were not included. Although these hospitals seldomly receive OHCA cases. However, the study revealed significant findings related to OHCA patients that can guide forthcoming research and national plans to advance the care of OHCA in Bahrain.

## Conclusion

The estimated annual incidence of OHCA attended by EMS was almost 21 per 100,000 population, with very low survival rates. The presence of low rate of bystander CPR, absent bystander AED, and prolonged operational defibrillation time by EMS mandate great attention to improve such factors. These issues need to be investigated in further studies. The study can guide officials to apply a bundle of interventions focused on community training for bystander CPR and AED, and to evaluate the current EMS approach for OHCA care. General community-level training for bystander CPR and AED should remain a keystone strategy. The country is in-need for OHCA registry for proper health surveillance in the community, quality improvement, and various observational research programs.

## CRediT authorship contribution statement

**Feras Husain Abuzeyad:** Writing – original draft, Visualization, Supervision, Project administration, Investigation, Conceptualization. **Yasser Chomayil:** Visualization, Supervision, Methodology, Investigation. **Moonis Farooq:** Visualization, Validation, Methodology, Investigation, Conceptualization. **Hamid Zafar:** Resources, Methodology, Data curation, Conceptualization. **Ghada Al Qassim:** Resources, Data curation. **Emad Minwer Saad Albashtawi:** Resources, Data curation. **Leena Alqasem:** Writing – review & editing, Conceptualization. **Naser Mohammed Ali Mansoor:** Resources, Data curation. **Danya Adel AlAseeri:** Resources, Data curation. **Ahmed Zuhair Salman:** Resources, Data curation, Conceptualization. **Muhammad Murad Ashraf:** Data curation, Resources. **Maryam Ahmed Shams:** Resources, Data curation. **Faisal Sami Alserdieh:** Resources, Data curation. **Mustafa Ali AlShaaban:** Resources, Data curation. **Abdulla Fuad Mubarak:** Resources, Data curation.

## Declaration of competing interest

The authors declare that they have no known competing financial interests or personal relationships that could have appeared to influence the work reported in this paper.
